# Study on Optimization of Liquid Fermentation Medium and Antitumor Activity of the Mycelium on *Phyllopora lonicerae*

**DOI:** 10.4014/jmb.2405.05004

**Published:** 2024-07-24

**Authors:** Min Liu, Lu Liu, Guoli Zhang, Guangyuan Wang, Ranran Hou, Yinghao Zhang, Xuemei Tian

**Affiliations:** 1 Shandong Province Key Laboratory of Applied Mycology, College of Life Sciences, Qingdao Agricultural University, Qingdao 266109, P.R. China; 2 College of Chemistry and Pharmaceutical Sciences, Qingdao Agricultural University, Qingdao266109, P.R. China

**Keywords:** *Phylloporia lonicerae*, liquid fermentation, anticancer activity, cell apoptosis, response surface methodology

## Abstract

*Phylloporia lonicerae* is an annual fungus that specifically parasitizes living *Lonicera* plants, offering significant potential for developing new resource food and medicine. However, wild resources and mycelium production of this fungus is limited, and its anti-tumor active ingredients and mechanisms remain unclear, hampering the development of this fungus. Thus, we optimized the fermentation medium of *P. lonicerae* and studied the anti-tumor activity of its mycelium. The results indicated that the optimum fermentation medium consisted of 2% sucrose, 0.2% peptone, 0.1% KH_2_PO_4_, 0.05% MgSO_4_·7H_2_O, 0.16% *Lonicera japonica* petals, 0.18% P fungal elicitor, and 0.21% *L. japonica* stem. The biomass reached 7.82 ± 0.41 g/l after 15 days of cultivation in the optimized medium, a 142% increase compared with the potato dextrose broth medium, with a 64% reduction in cultivation time. The intracellular alcohol extract had a higher inhibitory effect on A549 and Eca-109 cells than the intracellular water extract, with half-maximal inhibitory concentration values of 2.42 and 2.92 mg/ml, respectively. Graded extraction of the alcohol extract yielded petroleum ether phase, chloroform phase, ethyl acetate phase, and n-butanol phase. Among them, the petroleum ether phase exhibited a better effect than the positive control, with a half-maximal inhibitory concentration of 113.3 μg/ml. Flow cytometry analysis indicated that petroleum ether components could induce apoptosis of Eca-109 cells, suggesting that this extracted component can be utilized as an anticancer agent in functional foods. This study offers valuable technical support and a theoretical foundation for promoting the comprehensive development and efficient utilization of *P. lonicerae*.

## Introduction

*Phylloporia lonicerae* is an annual fungus that specifically parasitizes the stems of living *Lonicera* spp. and belongs to the Basidiomycota, Agaricomycetes, Agaricales, Hymenochaetaceae, and *Phylloporus* genera [1, 2]. Initially, the fungus was identified as *Phylloporia ribis* within the same genus. *P. ribis* primarily parasitizes plants of the genus Ribes and occasionally other plants. Consequently, *P. lonicerae* was considered as *P. ribis* that parasitec on *Lonicera* plants. In 2020, after research and demonstration, this fungus was formally classified as *P. lonicerae* [3]. Pharmacological studies have highlighted various beneficial properties of *Phylloporus* fungi, including anti-tumor [4-9], anti-diabetic [10], antioxidant [11, 12], immune-regulatory [13], and neurotrophic activities [14, 15]. These fungi are rich in secondary metabolites such as polysaccharides, sterols, triterpenes, polychlorinated compounds, and pyranones [16], which play crucial roles in their biological activities [17]. Furthermore, it shows promising results in treating pharyngitis and certain cancers [18].

The 2013 Announcement on Approval of 7 New Resource Foods by the Chinese Ministry of Health, which includes tea tree flowers, sanctioned the use of *P. lonicerae* fermentation mycelium as a new resource food. This approval provides significant policy support for the development and utilization of *P. lonicerae*. The fungus holds substantial potential in both food and medicine [4, 12, 18], with industrial production promising substantial economic benefits. However, the wild resources of this fungus are limited, and the yield of fermentation mycelium is low. Scientific research on its anti-tumor pharmacological activity mainly focuses on the anti-tumor aspects of polysaccharide and fruiting body. However, there are few studies on mycelium anti-tumor. These factors constrain the development and utilization of this fungal resource, with current utilization primarily confined to folk medicine and limited industrial production [19]. To overcome these challenges, this study optimized liquid fermentation culture medium and investigated the anti-tumor activity of *P. lonicerae*.

The addition of suitable herbal components to the liquid fermentation medium of medicinal fungi may regulate the growth metabolism of the fermentation process [20, 21]. Additionally, for parasitic fungi, host components may contribute to the fungus's growth metabolism. For instance, there are research reports that supplemented the culture medium of *Sanghuangporus Sanghuang* with an aqueous extract of mulberry branches, resulting in increased mycelial biomass during fermentation [22]. *P. lonicerae* thrives on *Lonicera japonica*, a traditional Chinese herbal medicine. Thus, in this study, we selected the petals and branches of *L. japonica* as eliciting factors to enhance the growth of *P. lonicerae*. Additionally, many fungal elicitors also regulate microbial growth and metabolism [23, 24]. Based on previous research in our laboratory demonstrating that P fungal elicitors (freeze-dried mycelium powder of *Perenniporia tenuis*) enhance fungal fermentation growth and active ingredient accumulation [25, 26], we included P fungal elicitors as eliciting factors to improve the fermentation growth of *P. lonicerae*.

Response surface methodology is a statistical approach that uses multiple quadratic regression equations to model the functional relationship between experimental factors and results. It addresses multivariate problems by analyzing these regression equations. Many optimizations of microbial fermentation process parameters have utilized this method [27-34]. Graded extraction and flow cytometry are commonly used for tracking active ingredients and studying tumor cell apoptosis [35-41]. Therefore, we optimized the liquid fermentation medium of *P. lonicerae* using response surface methodology and conducted preliminary investigations into the mechanism of anti-tumor effects through graded extraction and flow cytometry. Our study findings provide technical support and a theoretical basis for promoting the development and utilization of this fungal resource.

## Materials and Methods

### Fungal Materials and Cells

The wild *P. lonicerae* strain was isolated and preserved by the Key Laboratory of Applied Fungi in Shandong Province. Ovarian cancer cell line Skov3 (TCHu185), human non-small-cell lung cancer cell line A549 (TCHu150), Cervical cancer cell line Hela (TCHu187), Breast cancer cell line MCF-7 (TCHu74), and esophageal cancer cell line Eca-109 (iCell-h056) were purchased from the cell bank of the Chinese Academy of Sciences and Cybertron Biotechnology Co., Ltd.

### Reagents

The 3-(4,5-dimethylthiazol-2-yl)-2,5 diphenyl tetrazolium bromide (MTT), 5-fluorouracil, and apoptosis assay kits were purchased from Beijing Solarbio Technology Co., Ltd. (China). F-12K, McCoy's 5A, MEM, DMEM, RPMI-1640, and fetal bovine serum were purchased from Shanghai Xiaopeng Biotechnology Co., Ltd.(China). Phosphate-buffered saline buffer, penicillin-streptomycin-nystatin solution, and trypsin were purchased from Shanghai Genark Technology Co., Ltd. (China). *L. japonica* petal, P fungal elicitor, and *L. japonica* stem were self-made in the laboratory. Dimethyl sulfoxide solution was purchased from Tianjin Fuyu Fine Chemical Co., Ltd. (China) Petroleum ether, chloroform, ethyl acetate, and n-butanol were purchased from Xilong Science Co., Ltd. (China).

### Single Factor Climbing Experiment

Carrot, potato, sucrose, peptone, *L. japonica* petal, P fungal elicitor, and *L. japonica* stem were chosen as experimental factors. The experimental design is detailed in Table 1.

The basic culture medium used in the experiment contained glucose 2%, peptone 0.2%, KH_2_PO_4_ 0.1%, MgSO_4_·7H_2_O 0.05%, and distilled water, with a pH of natural. According to Table 1, the content of sucrose and peptone in the basic medium was changed respectively, and different solid medium was configured. According to Table 1, potato, carrot, *L. Japonica* stem, P fungal elicitor and *L. japonica* petal were added to the basic medium, and different solid media were configured. The inoculated plates were then incubated at 32°C in darkness for 30 days. The results were observed and analyzed post-culture.

### Response Surface Optimization

Design-Expert software (AtatEase, USA) was utilized for experiment design. The Box–Behnken method was employed to design a three-factor, three-level response surface optimization experiment. The liquid fermentation was conducted in 500 ml conical flasks with a 200 ml bottling capacity, with four groups of biological repeats. The flasks were subjected to shake flask fermentation conditions at 32°C and 120 rpm in darkness for 30 days. The results were observed and analyzed post-fermentation.

The same software was used for experimental design and data processing. Based on the optimization model, a liquid fermentation experiment was performed to verify the optimization results. A growth curve was plotted based on the fermentation experiment results.

### Preparation of Crude Extract of *P. lonicerae*

Activated *P. lonicerae* was transferred to liquid culture medium and shaken at 32°C and 120 rpm for 21 days, followed by expansion for cultivation. After cultivation, the mycelium was filtered and freeze-dried. Ten times the volume of 70% alcohol was added for ultrasound extraction. The supernatant was collected after centrifugation and this process was repeated twice. The three supernatants were combined, rotary evaporated, and freeze-dried to obtain an alcohol extraction sample (CT). Following alcohol extraction, a water extract (ST) was obtained using hot water.

### Preparation of Extraction Components from *P. lonicerae*

Fractional extraction was used for separation and purification, with petroleum ether, chloroform, ethyl acetate, and n-butanol selected for extraction. Next, 100 ml ultra-pure water was suspended with a 6 g alcohol extract sample, followed by the sequential addition of 200 ml petroleum ether, 200 ml chloroform, 300 ml ethyl acetate, and 300 ml n-butanol solvent for extraction. The extraction times were 3, 3, 6 and 3 times respectively, with each extraction lasting 1 h. The extraction liquid was concentrated to obtain petroleum ether phase, chloroform phase, ethyl acetate phase, and n-butanol phase successively. The samples were freeze-dried and stored in a 4°C refrigerator.

### Cell Culture and Treatment

Skov3, A549, Hela, MCF-7, and Eca-109 cells were respectively cultured in McCoy's 5A, F-12K, MEM, DMEM, RPMI-1640 medium with 10% fetal bovine serum as the complete medium at 37°C, 5% CO_2_, and 90% relative humidity. The cells were seeded into 96-well plates for culture. Five concentrations of 50, 10, 2, 0.4, and 0.08 mg/ml were selected, and the cells were treated with 100 μl of samples for 24, 48, 72, and 96 h, respectively, after which the sample solution was removed.

### Cell Proliferation Inhibition

MTT assay was used to determine the cell proliferation inhibition rate [42], with 5-fluorouracil as the positive control [43]; 20 μl of 5 mg/ml MTT was mixed with 80 μl of the corresponding complete medium, added to each well of the plate, and incubated at 37°C in a 5% CO_2_ incubator for 4 h. The mixed solution was removed, and 150 μl of dimethyl sulfoxide was added to dissolve the purple formazan crystals. The plate was shaken and mixed for 20 min, and the absorbance was measured at 490 nm. The inhibition rate was calculated using the formula:

Cell proliferation inhibition rate (%) = [1 − (A treated group/A control group)] × 100

### Cell Apoptosis Assay

During cell apoptosis, phosphatidylserine on the cell membranés inner surface will transfer to the outer surface, exposing phosphatidylserine externally. Annexin V easily binds to phosphatidylserine. Therefore, annexin V and propidium iodide double staining can be used to detect cell apoptosis by flow cytometry [44]. Eca-109 cells were seeded into six-well plates at a density of 1 × 10^5^ cells/ml and cultured to reach confluence. The experimental group was treated with petroleum ether samples of 200, 400, and 800 μg/ml sequentially, with a final volume of 2.5 ml per well. The control group received the corresponding volume of culture medium. After incubating in a carbon dioxide incubator for 48 h, the cells were processed according to the apoptosis detection kit and analyzed promptly using flow cytometry.

### Statistical Analysis

The half-maximal inhibitory concentration (IC_50_) of the sample solution was calculated and plotted using GraphPad Prism 9 statistical software (GraphPad Software Inc., USA). A significance level of *p* < 0.05 indicated statistical significance, while *p* < 0.01 indicated extremely significant statistical differences.

## Results

### Response Surface Optimization Results of *P. lonicerae* Liquid Fermentation Medium

**Fig. 1:**
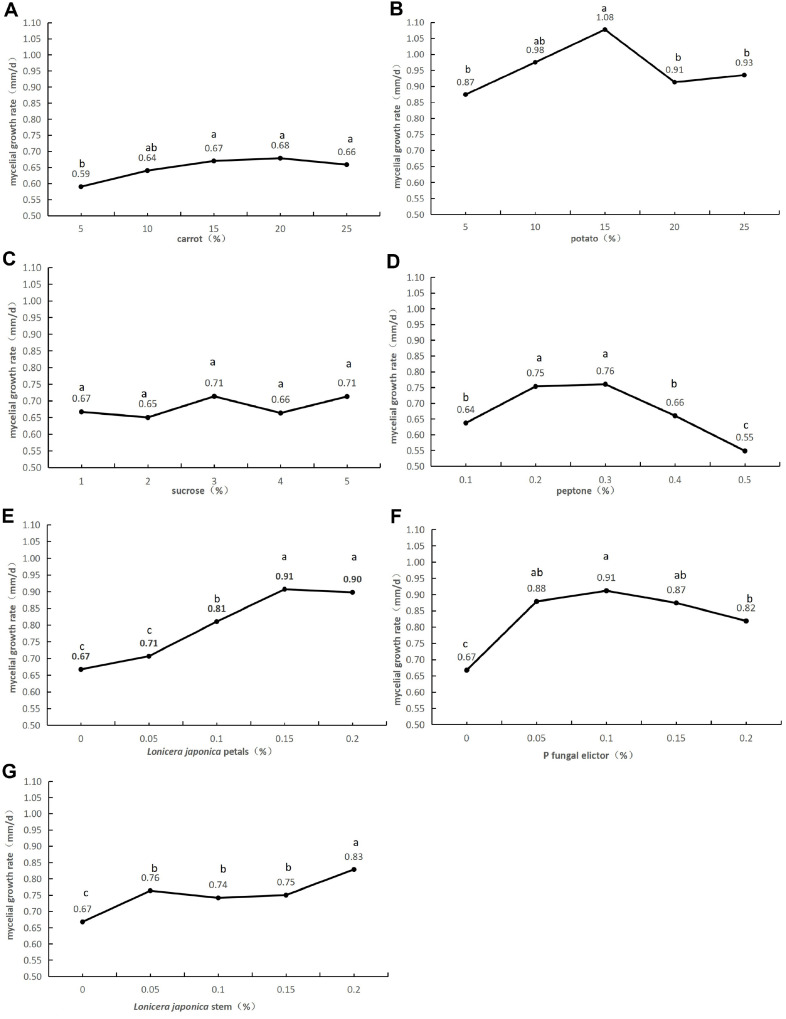
Effect of factors on the hyphal growth rate. Different letters indicate significant difference (*p* < 0.05), while similar letters indicate no difference.

**Single factor climbing experiment results.** As depicted in Fig. 1, the addition of carrot and sucrose had no significant effect on the mycelial growth rate. However, adding potatoes significantly promoted hyphal growth, with the most effective level at approximately 15%. Moreover, additions of peptone, *L. japonica* petals, P fungal elicitors, and *L. japonica* stems all significantly promoted mycelial growth, with optimal levels of approximately 0.2%–0.3%, 0.15%, and 0.1%, respectively.

**Response surface optimization results.** According to the results of preliminary experiments and single factor experiments, the formula of basic culture medium was glucose 2%, peptone 0.2%, KH_2_PO_4_ 0.1%, MgSO_4_·7H_2_O 0.05%, and distilled water, with a pH of natural. Box-Behnken response surface optimization design can significantly reduce the number of tests and thus reduce the test cost when the test factors are three. In addition, the selection of lower-priced medium components is more conducive to the promotion of large-scale production of *L. japonica*. Potato has a good effect on promoting mycelium growth, but its cost is higher. Moreover, compared with *L. japonica* petal, P fungal elicitor, and *L. japonica* stem, its significant influence on the results is not superior. Therefore, we only selected *L. japonica* petal, P fungal elicitor, and *L. japonica* stem, which are the most significant promoters of the growth of *P. lonicerae*, as the three influencing factors of Box-Behnken response surface optimization design. Mycelium biomass was used as the response in box-behnken desigens. *L. japonica* petal, P fungal elicitor, and *L. japonica* stem were selected for response surface optimization. Three factors and three levels box–behnken response surface experiment was designed using Design-Expert software (AtatEase). The experimental design and results are presented in Tables 2 and 3.

The experimental data were fitted by regression analysis, and the following second-order polynomial model was obtained.

Y = −2.02288 + 25.745 X1 + 47.23 X2 + 41.085 X3 − 55.25 X1X2 + 10.75 X1X3 − 55.5 X2X3 − 54.65 X1^2^ − 80.9 X2^2^ − 82.9 X3^2^ (1)

Y represents mycelium biomass, X1 represents *L. japonica* petal addition, X2 represents P fungal elicitor addition, and X3 represents *L. japonica* stem addition. The variance analysis of the fitting model is shown in Table 4.

The F value of the model was 14.870, and the P value was 0.001, which was less than 0.05, indicating that the model was accurate and reliable. The R^2^ value was 0.950, and the coefficient of variation was 6.40%, suggesting that the model can well reflect the test results. The predicted value of the model had a high correlation with the actual value. Among all the independent variables, interactive terms, and quadratic terms in this model, the model P values of X1, X2, X3, X1X2, X2X3, X12, X22, and X32 were less than 0.05, indicating that this model is significant.

**Fig. 2:**
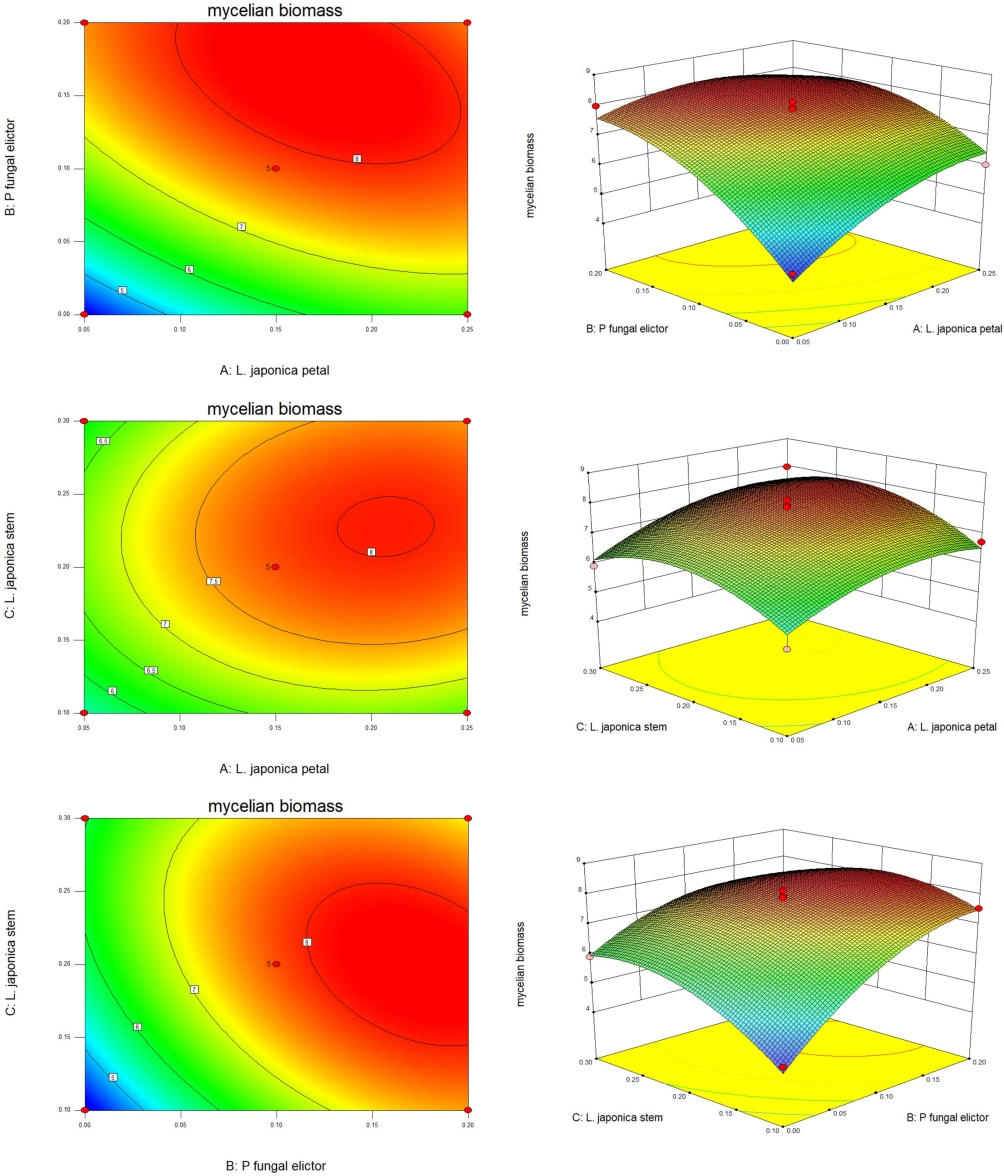
Contour map and response surface map of three-factor interaction. Response surface plots of the effects of P fungal elicitor and *L. japonica* petal on mycelial biomass, response surface plots of the effects *L. Japonica* stem and *L. japonica* petal on mycelial biomass; response surface plots of the effects *L. Japonica* stem and P fungal elicitor on mycelial biomass.

Fig. 2 displays the contour map and response surface map of the three-factor interaction. The oval contour map indicates a significant interaction among the three factors, suggesting that each factor can enhance the effect of the others at an optimal concentration. According to the simulated quadratic equation, when the amounts of *L. japonica* petal, P fungal elicitor, and *L. japonica* stem in the medium are 0.16%, 0.18%, and 0.21%, respectively, the maximum mycelium biomass can reach 8.22 g/l.

**Fig. 3:**
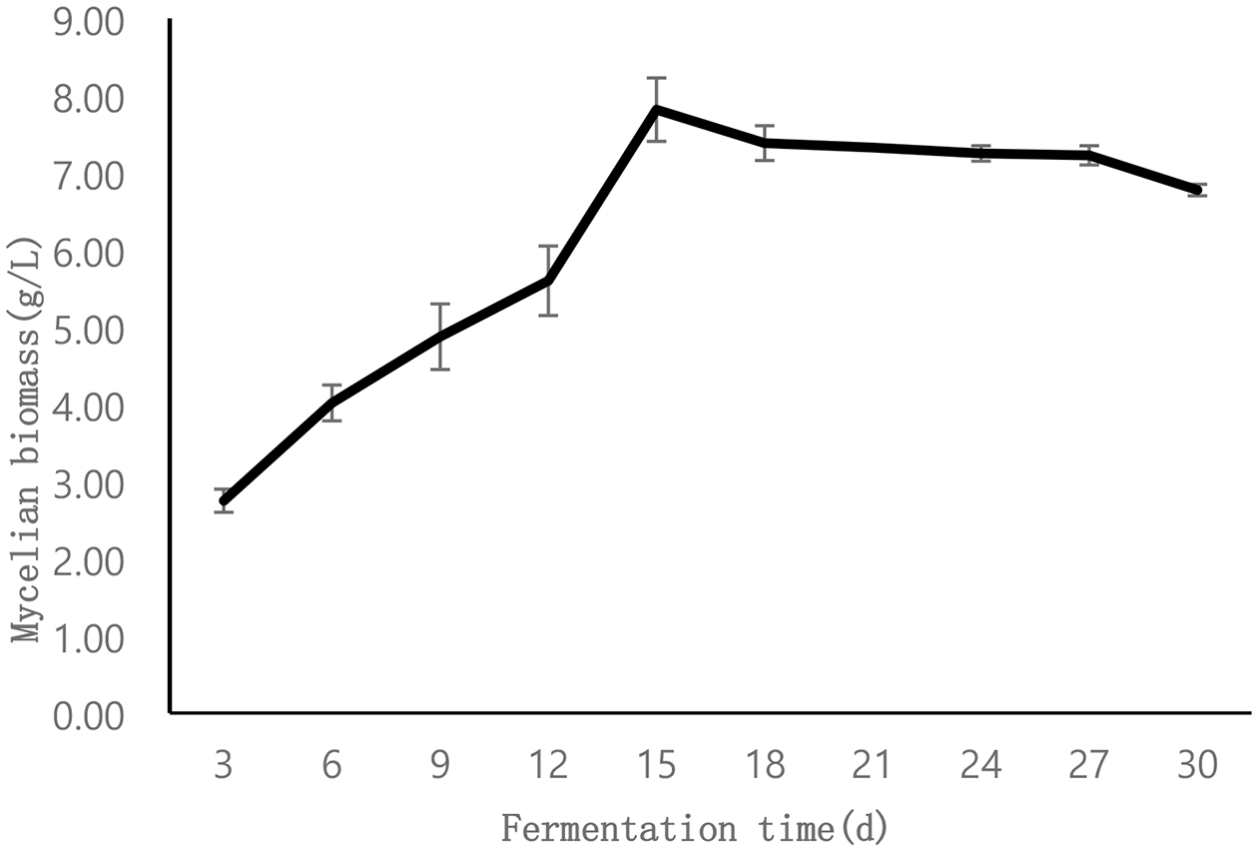
Fermentation growth curve of *P. lonicerae*. This figure showed the mycelial biomass changes of *P. lonicerae* cultured for 30 days.

**Fermentation verification test.** According to the response surface optimization model, the optimal fermentation medium for *P. lonicerae* consisted of 2% sucrose, 0.2% peptone, 0.16% *L. japonica* petal, 0.18% P fungal elicitor, 0.21% *L. japonica* stem, 0.1% KH_2_PO_4_, and 0.05% MgSO_4_·7H_2_O. When *P. lonicerae* was fermented with this optimized medium, the mycelium growth curve (Fig. 3) showed that the mycelium biomass reached a maximum value of 7.82 ± 0.41 g/l on the 15th day, which was not significantly different from the maximum value of 8.22 g/l predicted by the model. This shows that the difference between the results of verification test and the results predicted by the model is not statistically significant, and it can be considered that the results of verification test have reached the maximum of the statistical results of the model. In comparison, the biomass of the strain was 3.23 ± 0.9 g/l after 6 weeks' fermentation in potato dextrose broth medium. This study achieved a 64% reduction in culture time and a 142% increase in biomass.

### Research Results of Anti-Tumor Activity in *P. lonicerae*

**Fig. 4:**
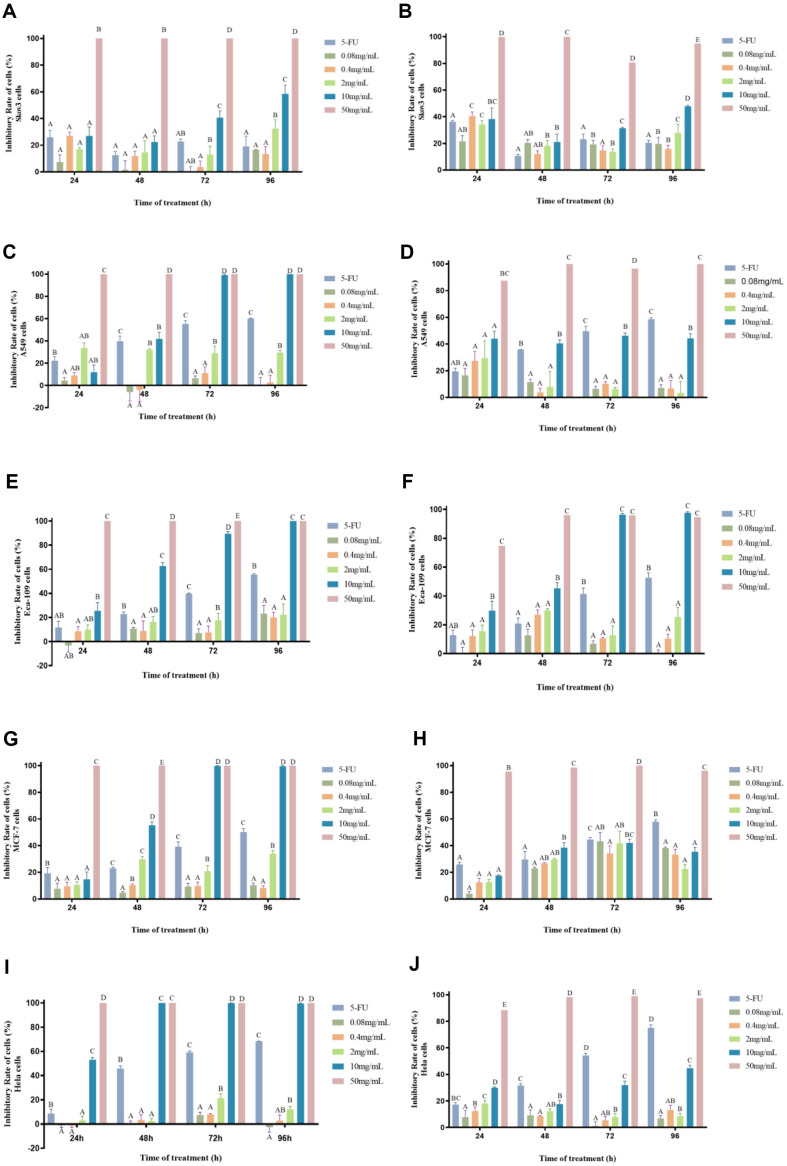
The cell proliferation levels of Skov3, A549, and Eca-109, MCF-7, Hela tumor cells after treatment with the crude extracts of *P. lonicerae* CT and ST at concentrations of 0.08, 0.4, 2, 10, and 50 mg/ml for 24, 48, 72, and 96 h. (**A, C, E, G, I**) represents the treatment of CT, and (**B, D, H, F, J**) represents the treatment of ST. 5-FU (200 μg/ml) was used as the positive control. Different letters indicate significant difference (*p* < 0.01), while similar letters indicate no difference.

**Tumor proliferation inhibition results of crude extract of *P. lonicerae*.** To explore the anti-tumor effects of CT and ST on various types of cancer cells, three cell lines (Skov3, A549, Eca-109, MCF-7, and Hela) were selected. CT and ST were tested at different concentrations (0.08, 0.4, 2, 10, and 50 mg/mL), and the results were assessed after 24, 48, 72, and 96 h (Fig. 4). The results indicate that the two samples have varying effects on the three types of cancer cells. As the concentration increased, CT and ST exhibited a dose-dependent inhibitory effect on the cancer cells, with the inhibition rate increasing to different extents over time. In Fig. 4A, CT showed no significant inhibitory effect on Skov3 cells at low concentrations. However, at higher concentrations, CT performed better than the positive control at 10 mg/ml, with an inhibition rate exceeding 50%. At 50 mg/ml, the sample showed a 100% inhibition rate at 24 h, significantly different from the positive control. On the other hand, ST demonstrated a notable inhibitory effect on Skov3 cells after 24 h (Fig. 4B), with an inhibition rate of 40.5% at 0.4 mg/ml, outperforming the positive control. The 50 mg/ml sample also achieved a 100% inhibition rate at 24 h. In Fig. 4C, CT showed no significant inhibitory effect on A549 cells at low concentrations. However, the inhibition rate of the 10 mg/ml sample significantly increased from 48 h to 72 h, reaching 100%, surpassing the positive 5-FU control. ST exhibited its highest inhibitory effect on A549 cells at 24 h (Fig. 4D), with no significant inhibition at low concentrations. The 50 mg/ml concentration achieved a 100% inhibition rate. The 10 mg/ml concentration had an inhibition rate of approximately 40%, which was not significantly different from the positive control. Regarding Eca-109 cells, the inhibition rate of CT at 10 mg/ml increased from 26% to 100% with prolonged treatment time (Fig. 4E), significantly different from the positive 5-FU control. At lower concentrations, the inhibition rates were around 20% or lower. For ST, the inhibition rate at low concentrations on Eca-109 cells was below 40% (Fig. 4F), but it increased to 100% at 10 mg/ml, indicating a certain inhibitory effect. In Fig. 4G, CT showed no significant inhibitory effect on MCF-7 cells at low concentrations. However, the inhibition rate of the 10 mg/ml sample significantly increased from 48 h to 72 h, reaching 100%. ST exhibited its highest inhibitory effect on MCF-7 cells at 72 h (Fig. 4H), the inhibition rate of low concentration samples was about 40%. CT performed better than the positive control at 10 mg/ml, and also achieved a 100% inhibition rate at 48 h. ST showed no significant inhibitory effect on Hela cells (Fig. 4J).

Table 5 presents the inhibitory effects of intracellular samples on different cell types. CT exhibited varying degrees of inhibition on the five cell types, showing a better effect on A549 cells, followed by Eca-109 cells. Similarly, ST demonstrated a better inhibitory effect on Eca-109 cells, with no significant additional inhibitory effect on A549 cells. Cell proliferation inhibition experiments using crude extracts of *P. lonicerae* indicated that CT had a positive effect on A549 and Eca-109 cells. After comparison, CT components have the potential to be further explored and utilized. Building on this result, CT was subjected to fractional extraction, and four organic phase samples were tested for *in vitro* anti-tumor experiments.

**Fig. 5:**
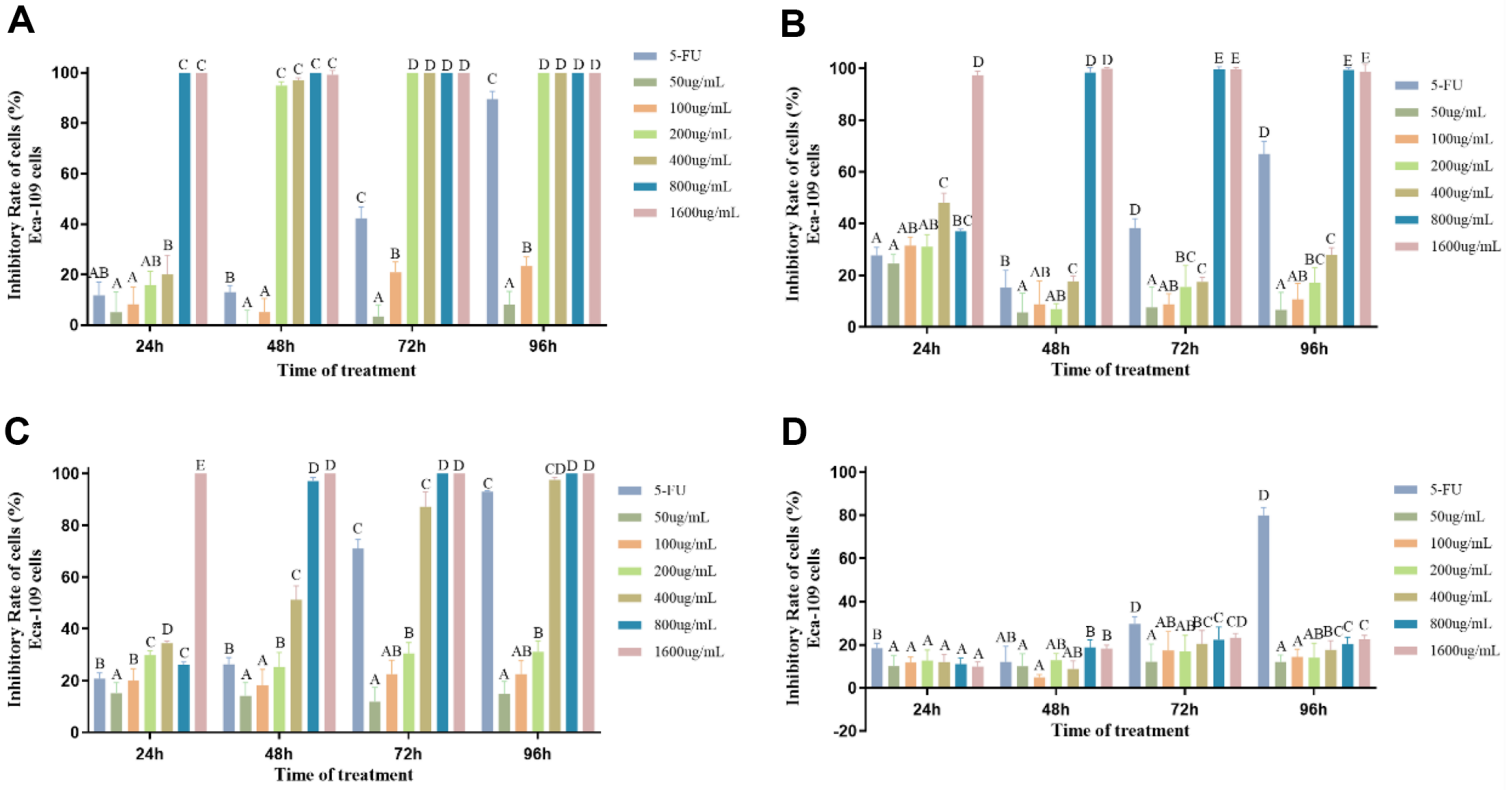
The cell proliferation levels of Eca-109 cells treated with extractive components for 24, 48, 72, and 96 h at extracted sample concentrations of 1600, 800, 400, 200, 100, and 50 μg/ml, respectively. (**A**) represents the treatment of petroleum ether samples, (**B**) represents the treatment of chloroform samples, (**C**) represents the treatment of Ethyl acetate samples, and (**D**) represents the treatment of N-butanol samples. 5-FU (200 μg/ml) was used as a positive control. Different letters indicate significant difference (*p* < 0.01), while similar letters indicate no difference.

**Tumor proliferation inhibition results of extracts from *P. lonicerae*.** Four components, namely petroleum ether phase, chloroform phase, ethyl acetate phase, and n-butanol phase, were obtained through extraction. Cell proliferation inhibition experiments were conducted on Eca-109 cells, with the four samples tested at different concentrations (1600, 800, 400, 200, 100, and 50 μg/ml). The results were assessed after 24, 48, 72, and 96 h (Fig. 5). As depicted in the figure, significant differences were observed in the inhibitory effects of the four samples on Eca-109 cells. The petroleum ether sample (Fig. 5A) exhibited a notable effect on cell proliferation. After 48 h of treatment, the proliferation inhibition rate of the 200 μg/ml sample exceeded 95%, with a stable effect observed, significantly differing from that of the positive control at the same concentration. The IC_50_ was calculated as 113.3 μg/ml (Table 6), indicating promising results. Samples with concentrations of 400, 800, and 1600 μg/ml achieved a 100% inhibition rate, with stable effects after 48 h of treatment. Conversely, the chloroform sample (Fig. 5B) showed a less pronounced effect on cell proliferation. While samples with concentrations of 800 and 1600 μg/ml exhibited notable effects, with inhibition rates reaching 100%, other concentrations showed rates below 30%, indicating an insignificant effect. In contrast, the ethyl acetate sample demonstrated a superior inhibitory effect on the cell line compared to the petroleum ether sample, with an IC_50_ of 229.2 μg/ml (Table 6). At low concentrations, the inhibition rates were all below 30%, with concentrations of 400 μg/ml showing rates of approximately 50% after 48 h of treatment, significantly differing from the positive control group. Samples with concentrations above 400 μg/ml showed promising effects, achieving a 100% inhibition rate.

**Fig. 6:**
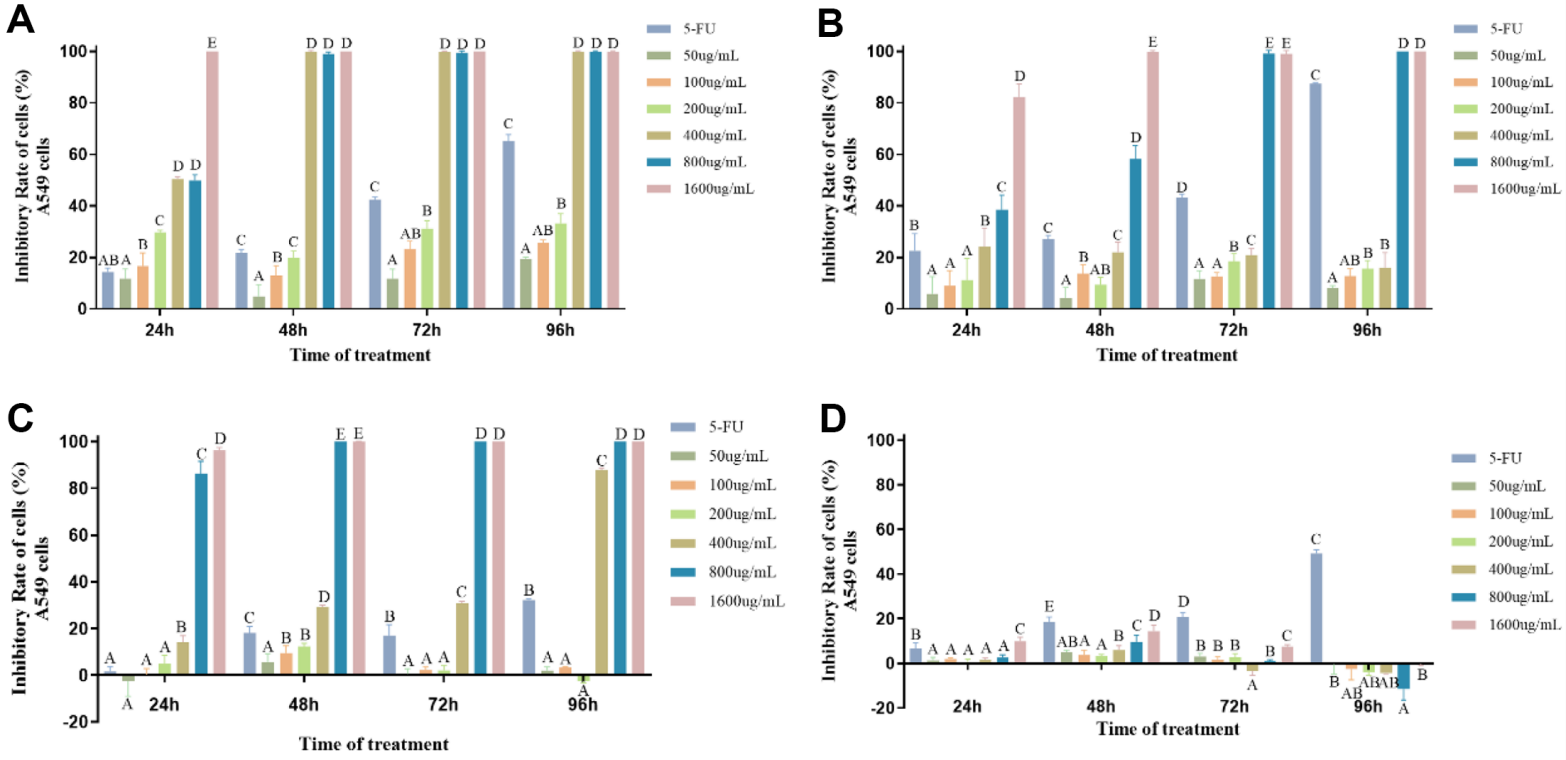
The cell proliferation levels of A549 cells treated with extractive components for 24, 48, 72, and 96 h at extracted sample concentrations of 1600, 800, 400, 200, 100, and 50 μg/ml, respectively. (**A**) represents the treatment of petroleum ether samples, (**B**) represents the treatment of chloroform samples, (**C**) represents the treatment of Ethyl acetate samples, and (**D**) represents the treatment of N-butanol samples. 5-FU (200 μg/ml) was used as a positive control. Different letters indicate significant difference (*p* < 0.01), while similar letters indicate no difference.

However, the n-butanol samples (Fig. 5D) showed no significant inhibitory effect on Eca-109 cells. Similar experiments conducted on A549 cells revealed significant differences in inhibition rates among the four samples. The petroleum ether sample demonstrated a relatively more significant effect on A549 cells, with a 100%inhibition rate observed after 48 h of treatment with a 400 μg/ml sample. At other low concentrations, the inhibition rates remained below 40% (Fig. 6), with no significant effect observed. The IC_50_ of the sample was calculated as 228.2 μg/ml (Table 6). In contrast, the chloroform and ethyl acetate samples exhibited less effective anti-tumor activity compared to the petroleum ether sample, with IC_50_ values exceeding 400 μg/mL. N-butanol samples showed no significant effect.

**Fig. 7:**
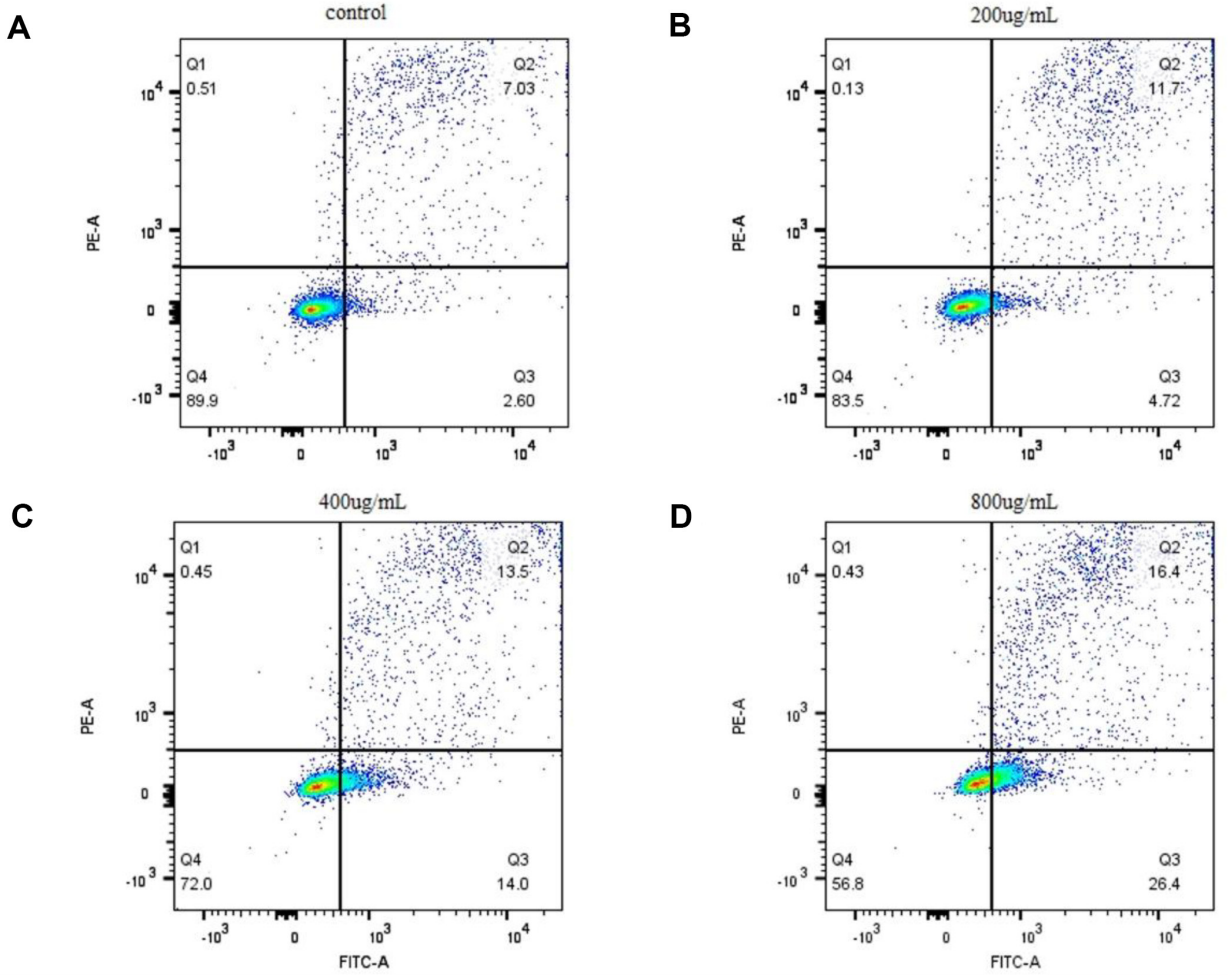
Effect of petroleum ether samples on apoptosis in Eca-109 cells treated with petroleum ether samples for 48 h based on Annexin V-FITC/PI staining. (**A**) represents the sample treated without petroleum ether, (**B**) represents the sample treated with 200 μg/ml petroleum ether, (**C**) represents the sample treated with 400 μg/ml petroleum ether, and (**D**) represents the sample treated with 800 μg/ml petroleum ether.

**Petroleum ether samples induced apoptosis in Eca-109 cells.** Apoptosis induction is a crucial mechanism of cell death triggered by anticancer drugs. In this study, early and late apoptosis of Eca-109 cells induced by petroleum ether components were detected using flow cytometry. Combining flow cytometry and detection results, it was observed that in the control group, most cells were alive, with a small percentage of dead cells (10.1%). However, after treatment with petroleum ether samples, the percentage of cells entering the apoptotic phase significantly increased, and the proportion of live cells decreased from 89.9% to 56.8%. Treatment with petroleum ether samples (200–800 μg/ml) led to a significant increase in the percentage of apoptotic cells in both the early (4.72%–26.4%) and late (11.7%–16.4%) stages (Fig. 7). These results were consistent with the findings of the MTT analysis, suggesting that the inhibitory effect of petroleum ether samples on the growth of Eca-109 cells was associated with the induction of apoptosis by these samples.

## Discussion

In this study, the experimental results revealed that the optimized medium for *P. lonicerae* included sucrose (2%), peptone (0.2%), KH_2_PO_4_ (0.1%), MgSO_4_·7H_2_O (0.05%), *L. japonica* petal (0.16%), P fungal elicitor (0.18%), and *L. japonica* stem (0.21%). The biomass of *P. lonicerae* reached 7.82 ± 0.41 g/l after 15 days of cultivation in the optimized fermentation system, a 142% increase compared with the potato dextrose broth medium, with a 64%reduction in cultivation time. Qin Guopei conducted related research on the liquid fermentation of *P. lonicerae* and suggested that glucose is the best carbon source for this process. His findings align with ours, indicating that small-molecule carbohydrate metabolism likely plays a crucial role in the respiratory metabolism of *P. lonicerae* [19]. Furthermore, Chow *et al*. also conducted a study where they added water extracts from the *L. japonica* stem to the *P. lonicerae* culture medium in an attempt to increase the liquid fermentation yield of the fungus. Their results indicated that the water extract of *L. japonica* stem significantly increased the content of ergosterol and total polysaccharides in the mycelium of the fungus, but did not have a significant promoting effect on mycelial biomass [45]. This differs from our findings and warrants further investigation.

In terms of *in vitro* anti-tumor effects, the analysis of cell inhibition by two crude extracts on three tumor cell lines revealed different inhibitory activities. The results showed that CT exhibited a significant inhibitory effect on A549 and Eca-109 tumor cells, while ST showed significant inhibition effects on Eca-109 cells and some inhibitory effect on Skov3 cells, but no significant inhibition on A549 cells. These findings suggest that *P. lonicerae* is an important medicinal fungal resource with the potential to develop products for treating non–small-cell lung cancer and esophageal cancer. The CT mainly contained terpenes, polyphenols, and flavonoids, while *P. lonicerae* contains betulinic acid, a triterpenoid compound with high pharmacological value [46]. Betulinic acid can inhibit the growth of human cervical cancer cells (HeLa) and breast cancer cells (MCF-7) while increasing the expression levels of p21 mRNA and p53 mRNA. This mechanism of action is associated with DNA damage in cells and the inhibition of cell cycle progression [47]. We also established that CT had a significant inhibitory effect on the proliferation of A549 and Eca-109 cells. Upon further component separation and extraction validations, it was evident that the petroleum ether sample exhibited a notably superior inhibitory effect on Eca-109 cells than 5-FU. Moreover, flow cytometry analysis demonstrated the ability of petroleum ether samples to induce cell apoptosis, thus impeding the growth of esophageal cancer cells. These results suggest that the intracellular alcohol extraction of petroleum ether components from *P. lonicerae* harbors potential anti-cancer agents.

## Figures and Tables

**Table 1:** Design of a single factor climbing experiment.

Factor/level	Carrot (%)	Potato (%)	Sucrose (%)	Peptone (%)	*L. japonica* petals(%)	P Fungal elctor(%)	*L.Japonica* stem(%)
1	5	5	1	0.1	0	0	0
2	10	10	2	0.2	0.05	0.05	0.05
3	15	15	3	0.3	0.10	0.10	0.10
4	20	20	4	0.4	0.15	0.15	0.15
5	25	25	5	0.5	0.20	0.20	0.20

**Table 2:** Response surface optimization design.

Factor/Level	L. japonica petal - X1(%)	P fungal elictor - X2(%)	L. japonica stem - X3(%)
1	0.05	0	0.1
0	0.15	0.1	0.2
-1	0.25	0.2	0.3

**Table 3:** Response surface optimization design and fermentation results.

Group	X1	X2	X3	Mycelium biomass Y (g/l)
1	0	1	-1	5.92 ± 1.35
2	1	1	0	4.38 ± 0.78
3	0	-1	1	7.53 ± 1.18
4	0	0	0	7.93 ± 0.47
5	-1	1	0	6.03 ± 0.51
6	0	0	0	7.73 ± 0.38
7	0	0	0	7.38 ± 0.02
8	0	1	1	4.25 ± 0.71
9	0	-1	-1	6.98 ± 3.58
10	1	0	1	5.10 ± 1.03
11	1	0	-1	5.92 ± 1.76
12	-1	0	-1	7.98 ± 0.08
13	1	-1	0	7.98 ± 0.08
14	0	0	0	8.12 ± 0.82
15	0	0	0	7.88 ± 0.52
16	-1	-1	0	7.42 ± 0.41
17	-1	0	1	6.73 ± 0.68

**Table 4:** Variance analysis of response surface fitting model.

Source	Type III Sum of Squares	Df	Mean Square	F Value	P-value
Model	25.190	9	2.800	14.870	0.001*
X1	2.860	1	2.860	15.180	0.006*
X2	10.880	1	10.880	57.820	0.000*
X3	1.270	1	1.270	6.760	0.035*
X1X2	1.220	1	1.220	6.490	0.038*
X1X3	0.046	1	0.046	0.250	0.635
X2X3	1.230	1	1.230	6.550	0.038*
X12	1.260	1	1.260	6.680	0.036*
X22	2.760	1	2.760	14.640	0.007*
X32	2.890	1	2.890	15.380	0.006*
Residual	1.320	7	0.190		
Lack of Fit	1.010	3	0.340	4.390	0.093
Puer Error	0.310	4	0.077		
Cor Total	26.510	16			

Df, degree of freedom; R^2^=0.950, CV=6.40%; *denotes signeficance level (*p* < 0.05) by F-test.

**Table 5:** IC_50_ of *P. lonicerae* on five types of cells.

Samples	A549 IC_50_ (mg/ml)	Eca-109 IC_50_ (mg/ml)	Skov3 IC_50_ (mg/ml)	MCF-7 IC_50_ (mg/ml)	Hela IC_50_ (mg/ml)
CT	2.42 ± 0.37	2.92 ± 0.18	4.65 ± 0.42	2.97 ± 0.33	2.99 ± 0.14
ST	-	2.97 ± 0.30	3.59 ± 0.48	3.34 ± 0.19	-

IC_50_ >10 mg/ml is recorded as "-".All experiments were performed in triplicate.Each value represents the mean ± SD (*n* = 3). n represents number of experiments.

**Table 6:** IC_50_ of the intracellular extracts of *P. lonicerae* on two cells.

Samples	Eca-109 IC_50_ μg/ml	A549 IC_50_ μg/ml
Petroleum ether	113.3 ± 0.55	228.2 ± 2.33
Chloroform	476.3 ± 0.80	478.8 ± 1.90
Ethyl acetate	229.2 ± 0.53	436.9 ± 1.56
N-butanol	-	-

IC_50_ >1000 μg/ml is recorded as "-".All experiments were performed in triplicate. Each value represents the mean ± SD (*n* = 3). n represents number of experiments.
